# Supplementary material for: When Research Evidence and Healthcare Policy Collide: Synergising Results and Policy into BRIGHTLIGHT Guidance to Improve Coordinated Care for Adolescents and Young Adults with Cancer
Source: Healthcare (Basel). 2025 Jul 26;13(15):1821. doi: 10.3390/healthcare13151821 (PMC12346042; doi:10.3390/healthcare13151821)
Supplement: Supplementary file 1 [file healthcare-13-01821-s001.zip › File S2.pdf]

THE POLICY INSTITUTE

KING'S College LONDON

Empowering stakeholders to shape implementation of the TYA cancer service specification

Policy Lab briefing pack

30 November 2022

About the BRIGHTLIGHT study and the follow-on project RELAY_ME

- BRIGHTLIGHT was an NIHR-funded national evaluation of specialist services for teenagers and young adults (TYA) with cancer and care delivered in Principal Treatment Centres (TYA-PTC)
- This model of care was proposed in 2005 by the NICE Improving Outcome Guidance
- The evaluation adopted a mixed-methods design, with a qualitative study exploring the culture of care and quantitative studies investigating the outcomes and costs associated with TYA-PTCs
- In the RELAY_ME project we are now working to communicate the findings from BRIGHTLIGHT, with the aim that they inform the design of specialist care services and improve outcomes for young people

What is a 'Policy Lab'?

- The Policy Institute at King's College London aims to bridge the gap between research, policy and practice using a variety of activities.
- A 'Policy Lab' is a focused, collaborative workshop bringing together a wide range of different stakeholders around a particular challenge to…
  - Assess the evidence
  - Understand barriers and constraints to change
  - Develop new ideas and practical approaches to improve outcomes

THE POLICY INSTITUTE

KING'S College LONDON

Structure of this briefing pack

1. Background and overarching question for the Policy Lab
2. Aims and agenda
3. Evidence from BRIGHTLIGHT to inform the day’s discussions
4. Involving stakeholders

1. Background and overarching question for the Policy Lab

The Policy Lab will address the following question

What is the roadmap for empowering different stakeholders to shape how the TYA service specifications are implemented?

The rationale for focusing on this is…

Situation

- Joint care – between specialist treatment centres and local hospitals – has been recommended in the TYA cancer service specification.
- This was based on the evidence available at the time of the specification's development (which was limited in relation to the outcomes of joint care).
- The service specification must now be implemented by a range of stakeholders.

Complication

- The BRIGHTLIGHT findings – published after the service specification was developed – showed that some outcomes were poorer for those receiving joint care.
- This has created a gap between the service specification and our understanding of what works well – a gap which needs to be bridged in its implementation.

Question

What is the roadmap for empowering different stakeholders to shape how the TYA service specifications are implemented?

2. Aims and agenda

Aims of the Policy Lab

The Policy Lab will bring together researchers, professionals from TYA cancer services, charities, policymakers and those with personal experience of the relevant issues to reflect on the project's findings and their implications for how stakeholders can work together to improve care.

The overarching question for the Policy Lab to address is:

What is the roadmap for empowering different stakeholders to shape how the TYA service specifications are implemented?

Our aim is to think broadly about the issues identified in the BRIGHTLIGHT study and their implications for how services are organised and delivered.

In doing so, we will encourage participants to think creatively about possible improvements, as well as the practicalities of implementing them at a suitable scale and the roles of different stakeholders in achieving this.

‘House rules’

1. The Policy Lab will be interactive, and engage participants in discussions and activities. The session will be fast-paced and collaborative to make the most of the range of experience within the group.
2. We want to create a relaxed, informal atmosphere where everyone feels comfortable to be honest and suggest ideas.
3. Given the limited time we have, we will have to stay focused on the overarching question set out in this pack and try to avoid tangents (however interesting!) as far as possible.
4. Discussions from the Lab will be captured in a number of ways, including in a publicly available output, but no quotes will be attributable directly to a particular individual.
5. We would like to take photographs during the session for communications about the project. If you would rather not be in any photographs, please let us know and we will make sure your face is not visible in any used.

Outputs

- Following the Policy Lab, discussions will be summarised in a **concise briefing note**, setting out some of the ideas discussed.
- This will be shared amongst the workshop participants and **disseminated widely** throughout the project's networks to raise awareness and encourage implementation.
- Any views of individuals expressed at the Policy Lab will not be included in a way that would make them identifiable.
- The discussions will also inform the **ongoing work** of the project team.

Agenda

**1000**	Welcome and introduction
	Reviewing the briefing pack
	Bridging the gap between the specification and evidence
	Identifying options for helping develop current ODN plans
**1300**	Lunch
**1345**	Developing a future roadmap for stakeholder contributions
	Measuring outcomes to track progress
	Next steps and thanks
**1600**	Close

Participant list

Research team

Rachel Taylor

Lorna Fern

Luke Hughes

Policy Institute

Alexandra Pollitt

Gabriel Lawson

Ross Pow

NHS England

Sue Osborne

Rachael Hough

Charities

Louise Soanes

National Cancer Research Institute

Dan Stark

NIHR

Martin Elliot

Young Advisory Panel

Amy Riley

Emily Freemantle

Steph Hammersley

Antonia Young

ODNs

Pia Fagelman

Maurice Brice

Toni Hunt

Andrew Davies

Jo Grout

Diane Hubber

Patrick Uriot

Hanna Simpson

Laura Elder

Liz Purnell

Amanda Saunders

Emma Thistlethwayte

Lisa Mcmonagle

3. Evidence from BRIGHTLIGHT to inform our discussions

Why there are separate services for teenagers and young adults?

- Teenagers and young adults (TYA) with cancer are a unique population with specific needs
- Outcomes including improvements in survival and participation in clinical trials are poorer than in younger children and older adults with similar cancers
- These unique circumstances have driven the development of care models specifically for teenagers and young adults with cancer, often focused on a dedicated purpose-designed patient environments supported by a multidisciplinary team (MDT) with specialised expertise
- In England, this model of care is commissioned by NHS England and delivered through 13 principal treatment centres (PTCs)

TYA Cancer Care Timeline

Key events relate to this workshop

- **1960s**: Establishment of distinct paediatric cancer services
- **1990**: The first teenage cancer unit opened at the Middlesex Hospital
- **2001**: Adult cancer services are reconfigured into cancer networks, resulting in improvements in both patient experience and outcomes
- **2005**: NICE publishes Improving Outcomes Guidance for children and young people with cancer, suggesting that it is ‘inappropriate’ to deliver care to young people in either child or adult settings
- **2012**: TYA networks of care are established, based around PTCs
- **2012:** Recruitment commences to the BRIGHTLIGHT Cohort
- **2019**: BRIGHTLIGHT whole programme of work presented at TYAC
- **202?**: TYA service specification released

Operational Delivery Networks

- Operational Delivery Networks are responsible for implementation of the service specification
- There are 13 TYA networks of care with a TYA-PTC and varying numbers of associated designated hospitals
- TYA-PTCs provide treatment expertise across the range of cancers common in young people, supported by a dedicated TYA multidisciplinary team (MDT)

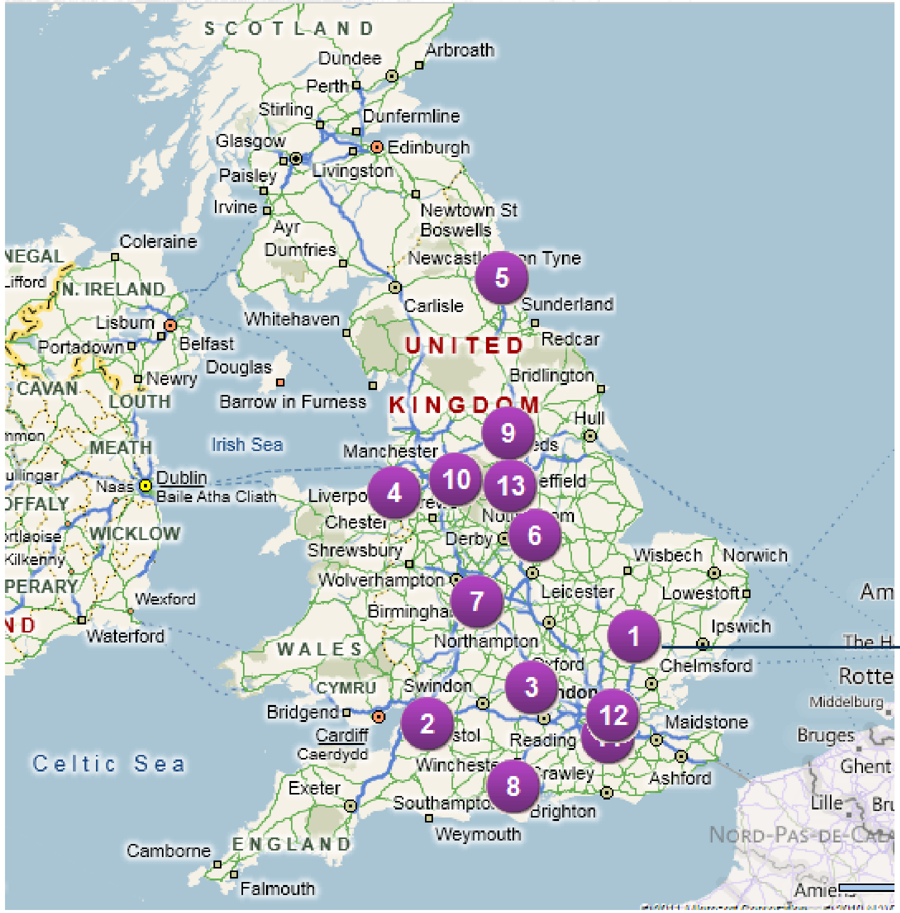

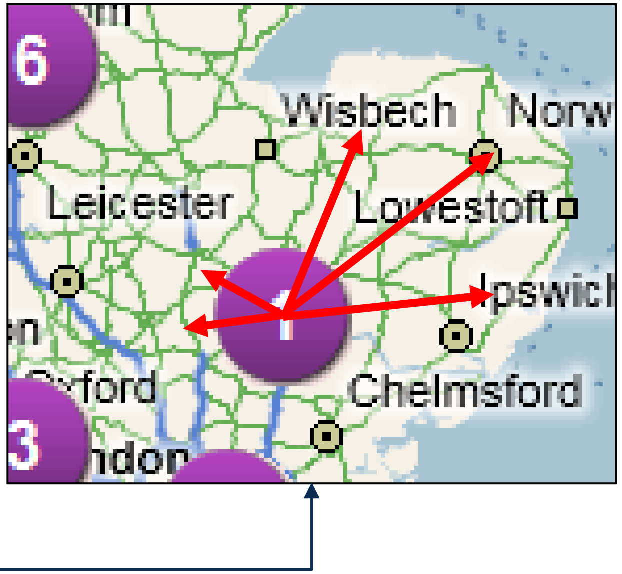

The BRIGHTLIGHT study

- Collected data that reflects TYA cancer services over the period 2012 to 2016.
- Headline findings relevant to the Policy Lab – presented on the following slides – relate to:
    - Treatment outcomes
    - Caregiver support
    - Expenses
    - TYA workforce and culture
- For further detail on the study, see: https://www.journalslibrary.nihr.ac.uk/pgfar/pgfar09120/#/abstract

Treatment outcomes

There are differences in outcome depending where young people are treated[1]

Young people who have…

- **no access to TYA-PTC** have:
  - higher quality of life (QoL) at diagnosis but a slower improvement in QoL over time
  - lower illness perception and lower reported processes associated with specialist care
- **all their care in a TYA-PTC** have:
  - the fastest rate of improvement in QoL
- **some care in a TYA-PTC** – i.e. some care in a specialist unit but also some care in a child or adult cancer unit – have:
  - the poorest QoL

Treatment outcomes - Survival

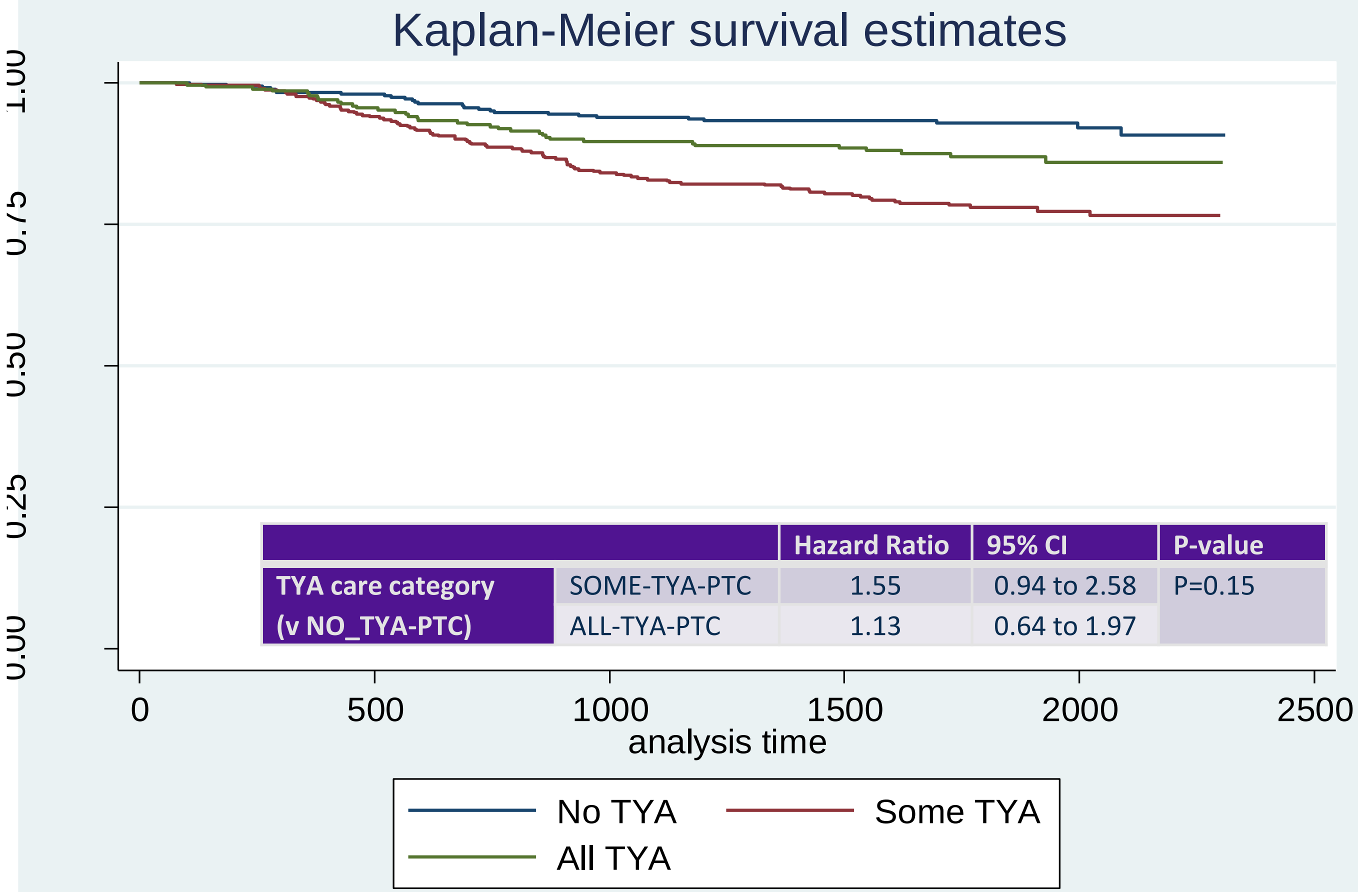

		Hazard Ratio	95% CI	P-value
TYA care category (v NO_TYA-PTC)	SOME-TYA-PTC	1.55	0.94 to 2.58	P=0.15
	ALL-TYA-PTC	1.13	0.64 to 1.97	

Young people who:

- had **no access to TYA-PTC** care had the highest survival,
- followed by those who received a**ll their care in a TYA-PTC**,
- followed by those who had **some care in a TYA-PTC**
- … but this is not statistically significant[2]

Treatment outcomes – Anxiety, Depression & Social Support

There was **no difference** in depression, anxiety or perceived social support between the three groups

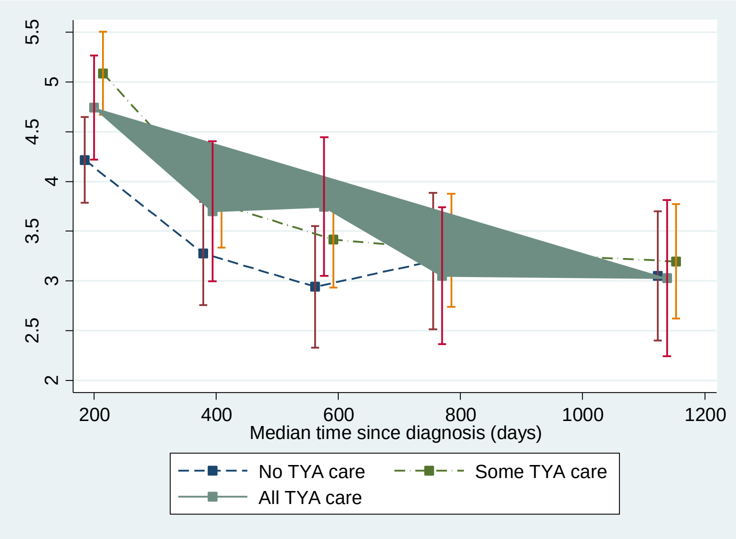

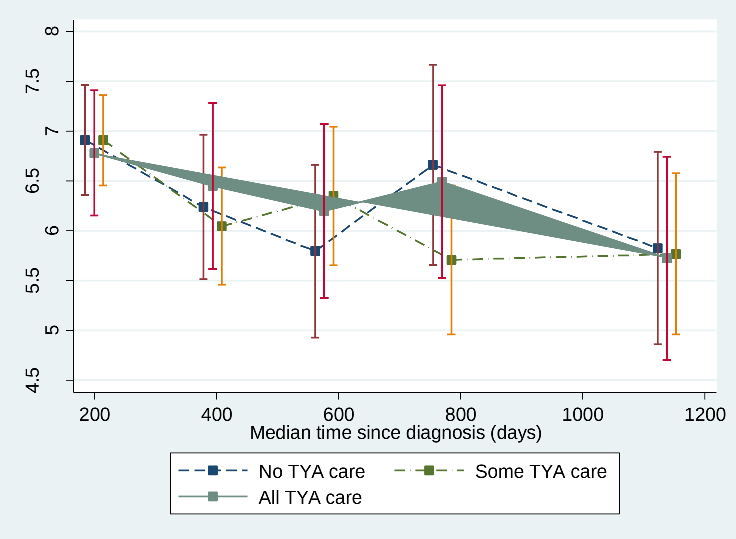

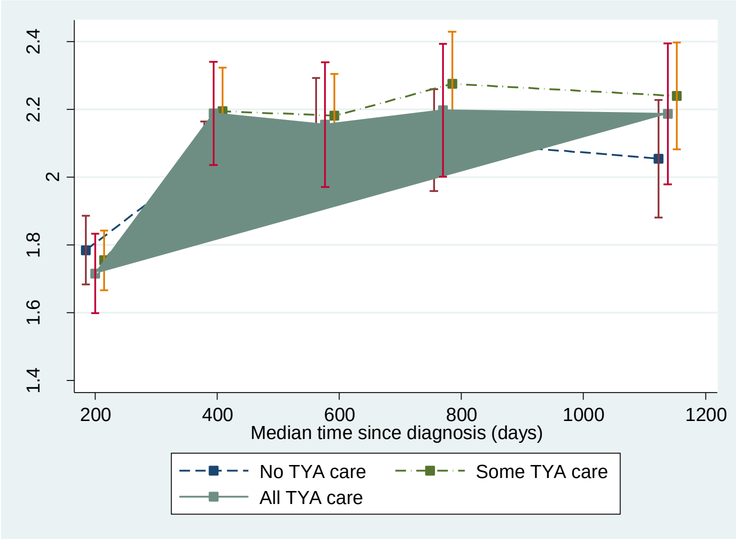

Treatment outcomes - Illness perception

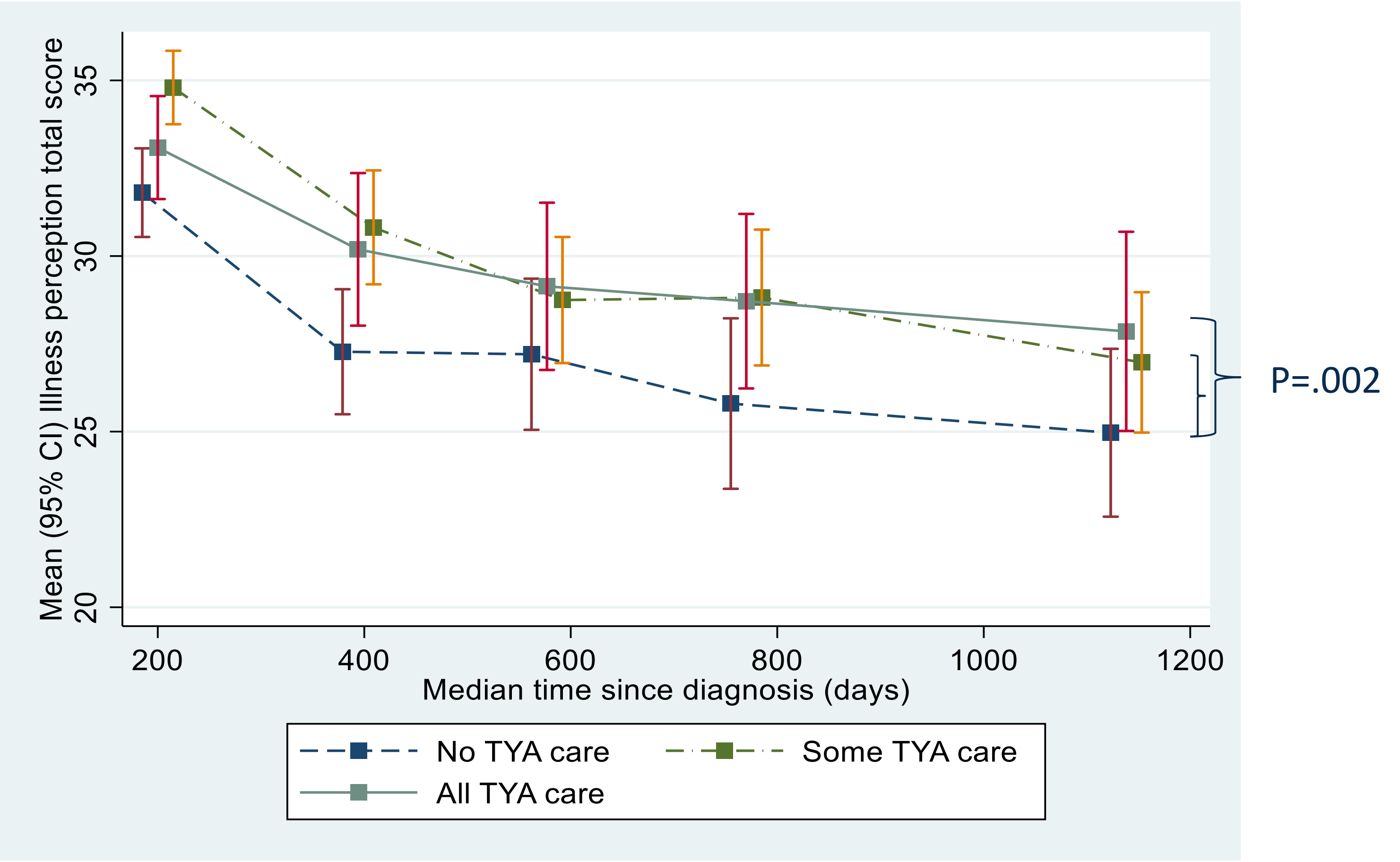

Young people who had **some** or **all** of their care delivered in a TYA-PTC had higher illness perception than those who received **no TYA-PTC care** (i.e. they were more likely to perceive themselves as ill)

Treatment outcomes - Process of care

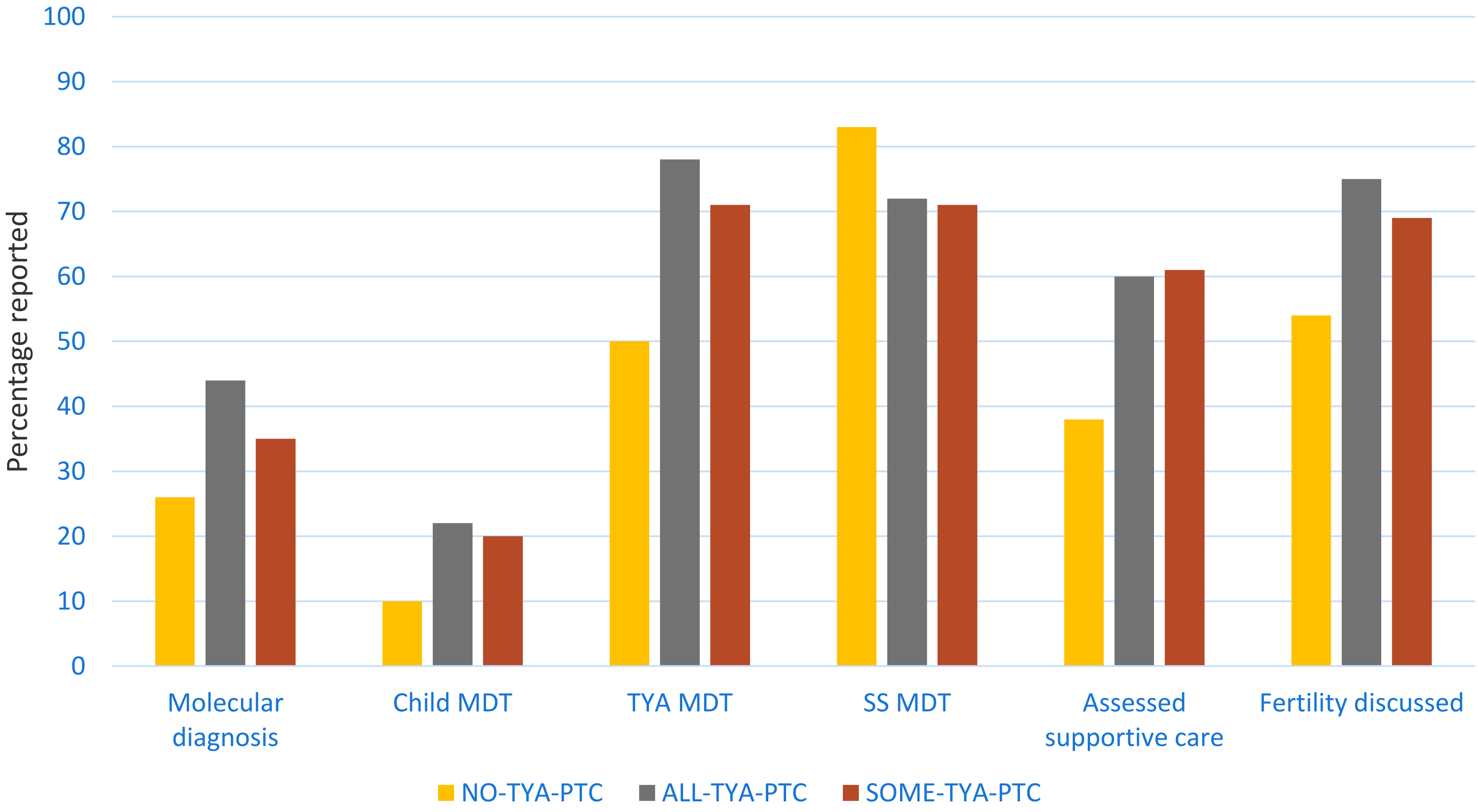

Young people receiving **no TYA-PTC care** were less likely to have a documented molecular diagnosis (where relevant), undergo a documented review by a children's or TYA MDT, have a documented assessment by supportive care services or receive a documented discussion regarding fertility[2]

Caregiver support

Again, there are differences in outcome depending where young people are treated[3]

Caregivers where young people have…

- **access to a TYA-PTC** (all or some care):
  - feel more supported
  - have greater access to information
  - feel there are more services specific for them
- **some care delivered by a TYA-PTC:**
  - feel less involved in decisions

Cost to the NHS

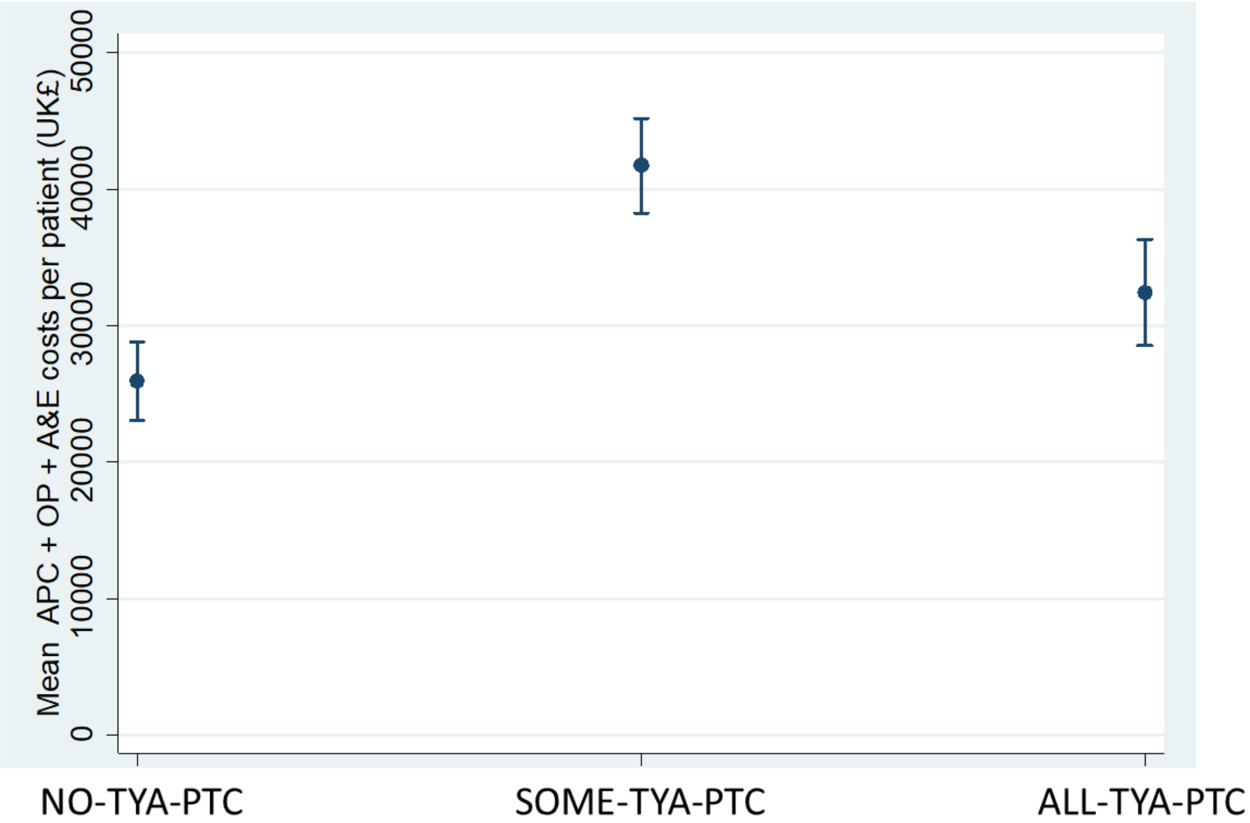

Some care in a TYA-PTC and some care elsewhere costs the most to the **NHS**, followed by **only TYA-PTC care**, followed by **no TYA-PTC care**

Cost to young people - travel

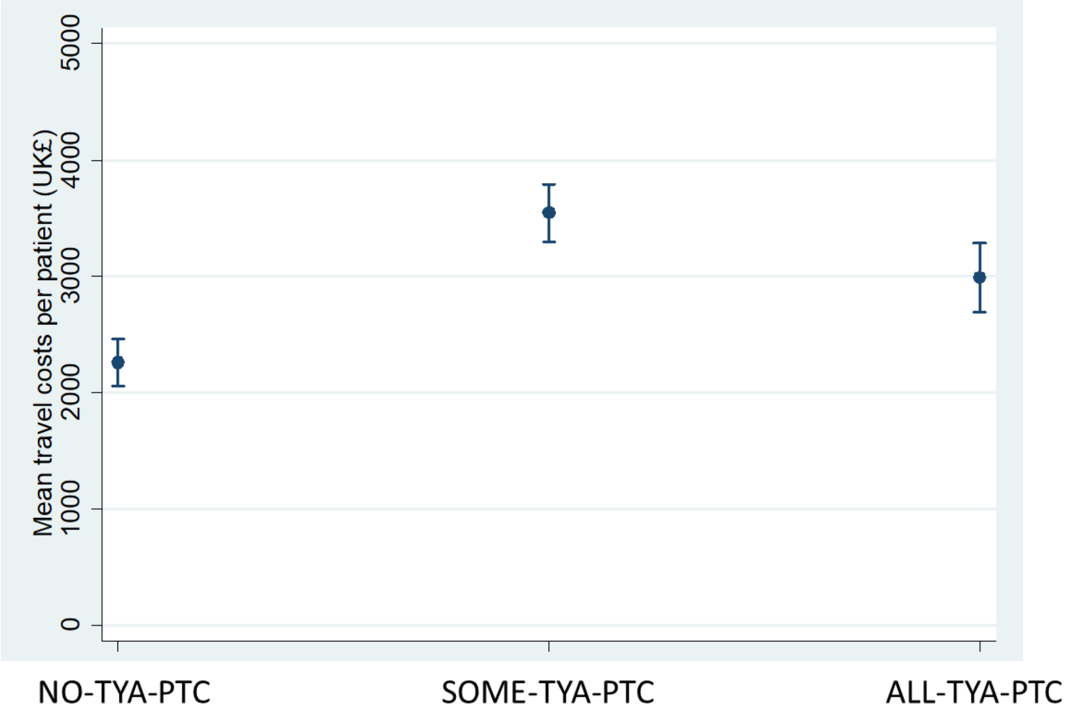

Category of TYA specialist care	n	Mean (£)	SE	95% CI	
NONE-TYA-PTC	733	2,204	100	2,008	2,400
SOME-TYA-PTC	733	3,510	125	3,264	3,755
ALL-TYA-PTC	733	2,950	150	2,656	3,245

Travel costs were greatest for **young people and their families** undergoing **some** care in a TYA-PTC

Out of pocket expenses

Out of pocket expenses (additional personal costs incurred because of a cancer diagnosis) were lowest for **young people** who had **no treatment in a TYA-PTC**

	NONE-TYA-PTC £ (n)	SOME-TYA-PTC £ (n)	ALL-TYA-PTC £ (n)
Calls	26.56 (86)	64.26 (97)	49.09 (59)
Food	101.76 (88)	480.39 (105)	293.33 (66)
Childcare	2.37 (95)	1.85 (108)	85.98 (61)
Respite Care	0 (95)	19.12 (113)	0 (66)
Pet Sitting/ Kennel Fees	1.07 (94)	10.51 (110)	15.82 (67)
Delivery/ Errand Charges	16.67 (95)	32.63 (107)	84.70 (66)
Cleaning Service	27.58 (95)	21.35 (111)	94.12 (68)
Domestic Help	2.87 (94)	14.44 (108)	58.15 (65)
Adaptations to Home	1.03 (97)	32.15 (107)	134.40 (67)
Clothing	41.15 (96)	83.12 (106)	69.55 (67)
Wig	13.46 (95)	48.60 (111)	14.78 (67)
Medical Equipment	3.02 (96)	8.95 (112)	5.30 (67)
Private Medical Fees	38.54 (96)	36.62 (111)	37.06 (68)
Counselling	2.08(96)	5.95 (111)	0 (67)
Physiotherapy	0 (96)	1.40 (114)	0 (68)
Total	284.77 (73)	743.83 (76)	976.46 (48)

Healthcare professional competence

- A distinct skill-set is needed in order to deliver high-quality TYA care
- Competence is required not just in cancer-related care but also in young person-related care[4]

Table 8 Top five areas of competence

Top five	Skill (n)	Knowledge (n)	Attitude (n)	Communication (n)
1	S7: Identify the impact of disease on young people's life (68; 50%)	K24: Know about side effects of treatment and how this might be different to those experienced by children or older adults (65; 48%)	A7: Honesty (84; 62%)	C8: Listen to young people's concerns (90; 66%)
2	S26: Have excellent clinical skills (53; 39%)	K18: Know how to provide age-appropriate care (55; 40%)	A1: Friendly and approachable (64; 47%)	C9: Talk about difficult issues (86; 63%)
3	S17: Work in partnership with young people (52; 38%)	K19: Know about current therapies (50; 37%)	A20: Be committed to caring for young people with cancer (58; 43%)	C15: Speak to young people in terms that are familiar to them while retaining a professional boundary (76; 56%)
4	S29: Able to discuss sensitive subjects, eg, sexual issues, fertility (43; 32%)	K23: Know about impact of cancer on psychological development (42; 31%)	A11: Be respectful (54; 40%)	C2: Tell young people about all aspects of their disease (63; 46%)
5	S11: Deliver patient-centred care (39; 29%)	K3: Developmental issues related to emerging adulthood (41; 30%)	A25: Ability to use humour appropriately when interacting with young people (52; 38%)	C1: Act as an advocate for young people (59; 43%)

Defining age-appropriate care

- Care delivered in an environment that promotes normality is essential to the delivery of optimal holistic and young person-centred care
- Creating this culture of care takes time and commitment[5]

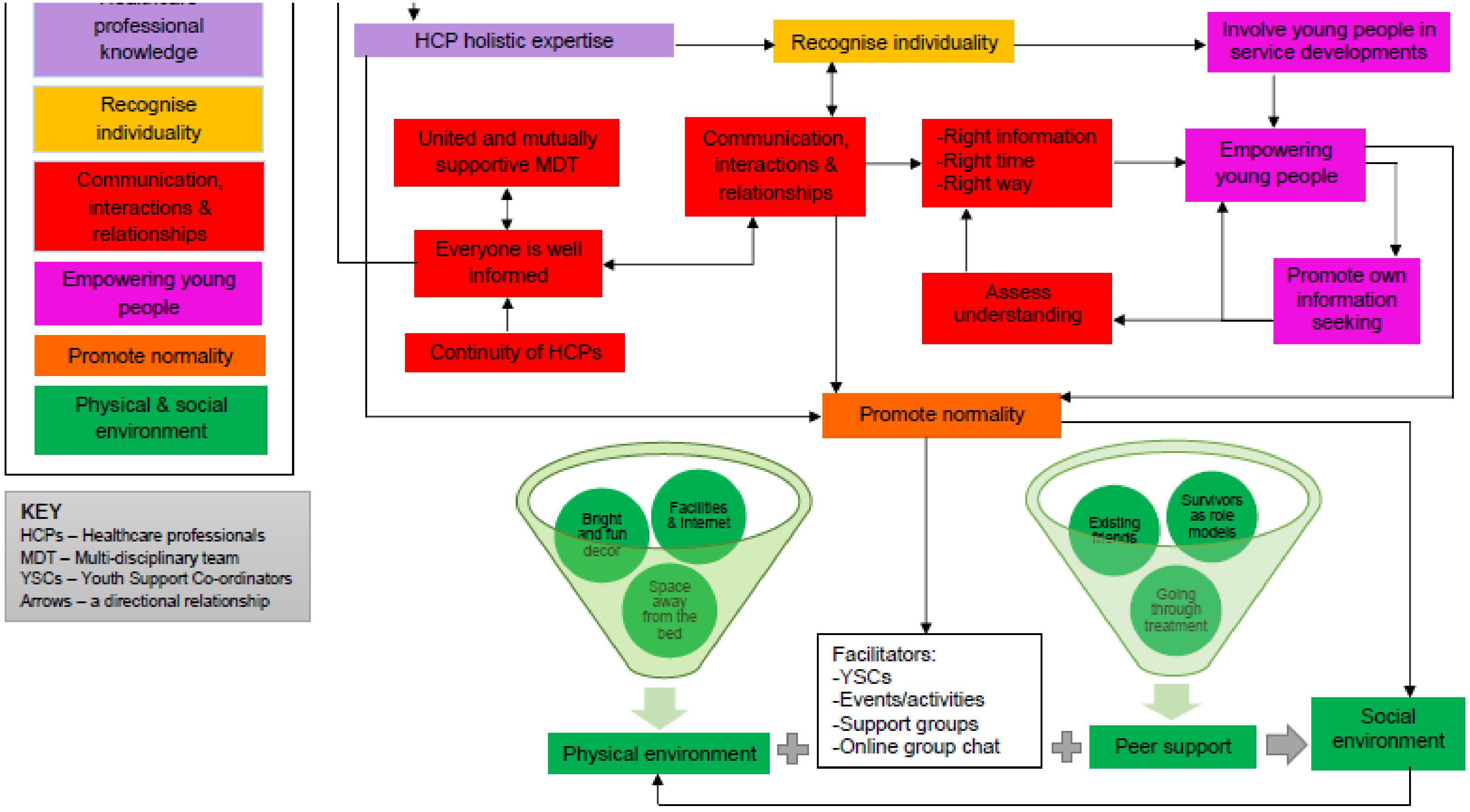

The unique culture of TYA Care

The formation of a culture responsive to the unique needs of teenagers and young adults is influenced by four factors:

- a consistent volume of young people using services
- effective leadership
- shared beliefs and 'buy in' from healthcare professionals
- professional holistic competence among healthcare professionals[6]

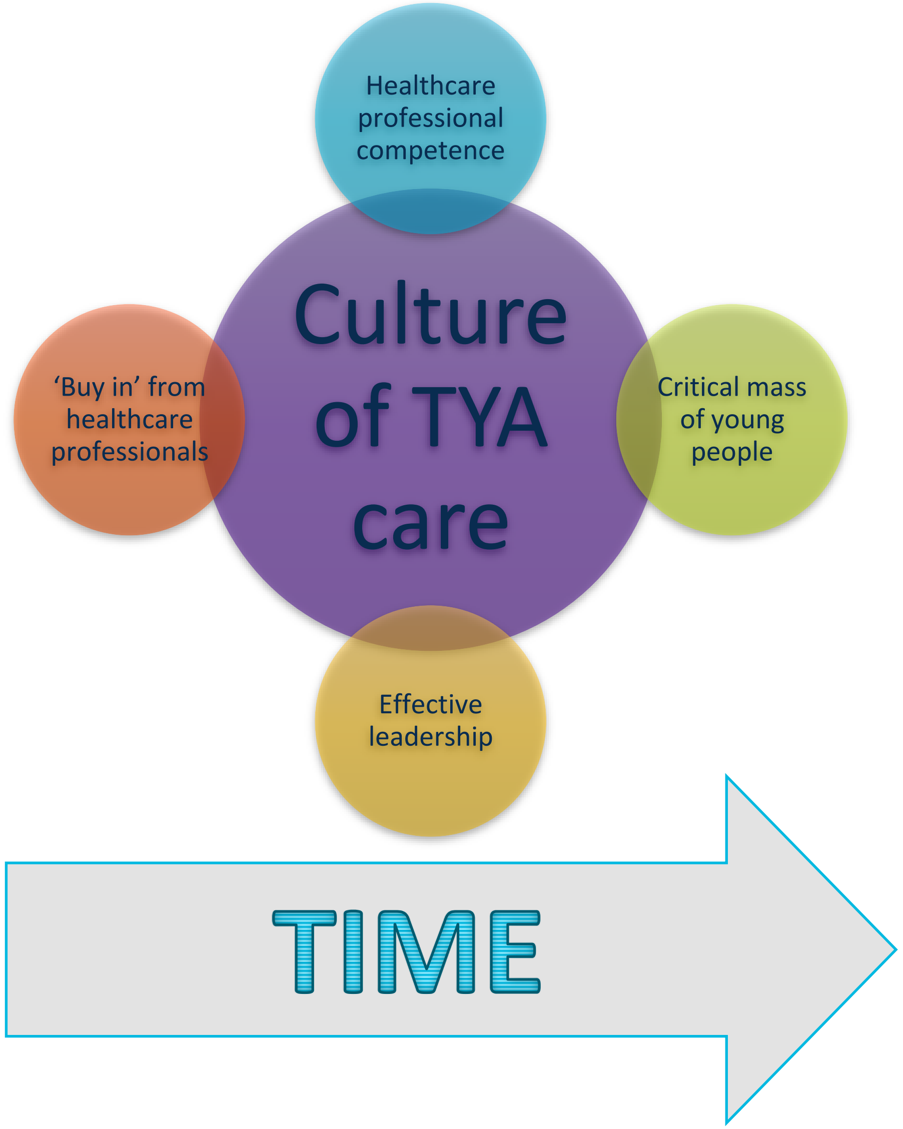

4. Involving stakeholders…

What was our Young Advisory Panel's interpretation of the results?

- Three years follow up is not be long enough to capture the impact of being treated in TYA-PTC
  - 5 and 10 year follow up would be better
- The scale needs to be adjusted to measure the amount of time spent in TYA-PTC versus non-TYA-PTC
- Do certain cancers benefit more from being in the TYA-PTC?
- Excess costs in SOME-TYA-PTC groups could be explained by duplicate tests
- The diagnostic experience is also important – especially costs to the young person and family.

Who are the key stakeholders in effective implementation?

- Young people
- Members of their social support network
- Members of the MDT in both PTC and designated hospitals
- Community and primary care
- Third sector
- Commissioners

References

1. Taylor RM, Fern LA, Barber JA, Alvarez-Galvez J, Feltbower R, Lea S, Martins A, Morris S, Hooker L, Gibson F, Raine R, Stark DP, Whelan JS. (2020) Specialist age-appropriate care and quality of life outcomes in a longitudinal cohort of teenagers and young adults: the BRIGHTLIGHT study. *BMJ Open* e038471. doi:10.1136/bmjopen-2020-038471
2. Fern LA, Taylor RM, Barber JA, Alvarez-Galvez J, Feltbower R, Lea S, Martins A, Morris S, Hooker L, Gibson F, Raine R, Stark DP, Whelan JS. (2021) Processes of care and survival associated with treatment in specialist teenage and young adult cancer centres: results from the BRIGHTLIGHT cohort study. *BMJ Open* doi:10.1136/bmjopen-2020-044854
3. Martins A, Alvarez-Galvez J, Fern LA, Vindrola C, Gibson F, Whelan JS, Taylor RM. (2019) The BRIGHTLIGHT national survey of the impact of specialist teenage and young adult cancer care on caregivers' information and support needs. *Cancer Nursing* DOI: 10.1097/NCC.0000000000000771
4. Taylor RM, Feltbower RG, Aslam N, Raine R, Whelan JS, Gibson F. (2016) A modified international e-Delphi survey to define healthcare professional competencies for working with teenagers and young adults with cancer. *BMJ Open* 6:e011361. doi:10.1136/bmjopen-2016-011361
5. Lea S, Taylor RM, Martins A, Fern LA, Whelan JS, Gibson F. (2018) Conceptualising age-appropriate care for teenagers and young adults with cancer: a qualitative mixed methods study. *Adolescent Medicine Health and Therapeutics*. 9: 149-166 doi: 10.2147/AHMT.S182176
6. Lea S, Taylor RM, Gibson F. (2022) "It all clicks together in the end": developing, nurturing and sustaining a teenage and young adult-centred culture of care. *Qualitative Health Research* doi: 10.1177/10497323221084910s

THE POLICY INSTITUTE

Connect with us

@policyatkings www.kcl.ac.uk/sspp/policy-institute
